# Heat Shock Protein-70 (Hsp-70) Suppresses Paraquat-Induced Neurodegeneration by Inhibiting JNK and Caspase-3 Activation in *Drosophila* Model of Parkinson's Disease

**DOI:** 10.1371/journal.pone.0098886

**Published:** 2014-06-02

**Authors:** Arvind Kumar Shukla, Prakash Pragya, Hitesh Singh Chaouhan, Anand Krishna Tiwari, Devendra Kumar Patel, Malik Zainul Abdin, Debapratim Kar Chowdhuri

**Affiliations:** 1 Embryotoxicology Section, CSIR-Indian Institute of Toxicology Research, Lucknow, Uttar Pradesh, India; 2 School of Biological Sciences and Biotechnology, Indian Institute of Advanced Research, Gandhinagar, Gujrat, India; 3 Analytical Section, CSIR-Indian Institute of Toxicology Research, Lucknow, Uttar Pradesh, India; 4 Academy of Scientific and Innovative Research, New Delhi, India; 5 Department of Biotechnology, Jamia Hamdard, New Delhi, India; CSIR-Central Drug Research Institute, India

## Abstract

Parkinson's disease (PD) is one of the most common neurodegenerative disorders with limited clinical interventions. A number of epidemiological as well as case-control studies have revealed an association between pesticide exposure, especially of paraquat (PQ) and occurrence of PD. Hsp70, a molecular chaperone by function, has been shown as one of the modulators of neurological disorders. However, paucity of information regarding the protective role of Hsp70 on PQ-induced PD like symptoms led us to hypothesize that modulation of *hsp70* expression in the dopaminergic neurons would improve the health of these cells. We took advantage of *Drosophila*, which is a well-established model for neurological research and also possesses genetic tools for easy manipulation of gene expression with limited ethical concern. Over-expression of *hsp70* was found to reduce PQ-induced oxidative stress along with JNK and caspase-3 mediated dopaminergic neuronal cell death in exposed organism. Further, anti-apoptotic effect of *hsp70* was shown to confer better homeostasis in the dopaminergic neurons of PQ-exposed organism as evidenced by their improved locomotor performance and survival. The study has merit in the context of human concern since we observed protection of dopaminergic neurons in PQ-exposed organism by over-expressing a human homologue of *hsp70, HSPA1L,* in these cells. The effect was parallel to that observed with *Drosophila hsp70.* These findings reflect the potential therapeutic applicability of *hsp70* against PQ-induced PD like symptoms in an organism.

## Introduction

Neurons, the building blocks of the nervous system, are reported to have limited regeneration capability after damage [1]. Thus, progressive loss in structure or function of neurons can result in various neurodegenerative disorders [2]. These neurodegenerative processes have been associated with a number of diseases in humans such as Parkinson's-, Alzheimer's-, Huntington's-disease, etc. Among them, Parkinson's disease (PD) has been described as the second most common progressive movement disorder [3]. It is characterized by the loss of dopaminergic neurons within the substantia nigra region of the midbrain that leads to problem in walking and difficulty in maintaining balance [4].

The multifactorial etiology of PD has been linked to aging, genetic and environmental factors [5]. However, earlier reports, including epidemiological findings [6]–[8] emphasized that environmental factors play major role in the pathogenesis of PD. Among the environmental factors, paraquat (PQ), a widely used herbicide, has been shown to produce PD like symptoms in exposed organisms [6], [9]. This association is further supported by higher PD incidences in the population with occupational exposure to PQ [8]. Moreover, generation of oxidative stress (OS) and subsequent activation of JNK and caspase-3 mediated death of dopaminergic neurons was revealed as one of the underlying mechanisms of PQ-induced PD [7]. Since, PQ toxicity is mediated through OS, efforts have been made to diminish such negative impact by using various anti-oxidants such as superoxide dismutase (SOD), Coenzyme Q10 [10], [11] etc.

Heat shock protein 70 (Hsp70), a key molecular chaperone [12], with a functional analogy to an anti-oxidant, is reported to protect cells from oxidative damage [13]. In general, heat shock proteins (HSPs) act as molecular chaperones that assist in the correct folding of nascent and stress-accumulated mis-folded proteins and prevent their aggregations [14]. Our laboratory has shown *hsp70* expression as the first-tier bio-indicator of chemical induced toxicity since this gene was found to be the first inducible gene in the organism after chemical stress [15], [16]. Moreover, it has also been reported as a negative regulator of apoptosis in an organism as it modulates apoptosis inhibiting factor (AIF), caspase-3 and others [17], [18].

Besides the defensive role of Hsp70 in OS, the former is also suggested as a potential therapeutic target for the treatment of neurological diseases [12], [19]. For example, protective role of Hsp70 in α-synuclein (αSN) induced toxicity was shown in different models, including *Drosophila* [19]–[21]. In addition, *in vitro* and *in vivo* studies have demonstrated that geldanamycin, valproic acid and celastrol induced *hsp70* expression can rescue neurotoxicity caused by 1-methyl-4-phenyl-1,2,3,6-tetrahydropyridine (MPTP) and rotenone [21]–[23]. However, these studies had limitations since the above mentioned inducers are reported to produce side effects after prolonged usage [12], [24]. In this context, Samuni et al. [24] have reported hepatotoxicity after the use of geldanamycin and its analogues in rat primary hepatocytes. Considering the above, genetic manipulation of *hsp70* may be a viable option to achieve protection against chemical induced neurodegenerative disease like conditions. Except for an *in vitro* study, where *hsp70* over-expression was shown to intervene PQ-induced neurotoxicity in rat neuroblast cells [25], no *in vivo* study has been reported so far on the direct role of Hsp70 in alleviating PQ-induced PD like symptoms. Therefore, we hypothesized that over-expression of *hsp70* in the dopaminergic neurons of an organism can protect it against PQ-induced PD like symptoms.

In order to address the above, we used *Drosophila melanogaster* which is a well-established model organism for studying human neurodegenerative disorders [19], [26] including PQ-induced PD [6]. Here, we over-expressed *hsp70* (both *Drosophila* and its human homologue) in the dopaminergic neurons of the flies by using a UAS/Gal4 system [27] and explored the protective role of Hsp70 against PQ-induced PD like symptoms in exposed organism.

## Materials and Methods

### *Drosophila* culture and PQ exposure

Fly stocks *w^1118^*, *Df(hsp70), TH-Gal4, Hsp70K71E* (a dominant negative mutant of *hsp70*, which results in compromised expression of endogenous *hsp70* after driving with Gal4) [28], *UAS-hsp70* (results in the over-expression of *hsp70* after driving with Gal4), and *HSPA1L* (human homologue of Hsp70) [29] were used. Flies were reared on standard *Drosophila* food [30] at 24±1°C. *TH-Gal4* strain was used to modulate the expression of *hsp70* in the dopaminergic neurons of *Drosophila. w^1118^* and *TH-Gal4>w^1118^* were used as genetic control against *hsp70* deficient strain and strains having genetic modulation of *hsp70*, respectively.

The flies were exposed to PQ according to Girardot et al [31] with minor modifications, using 5% sucrose and 1.3% agar as the medium. Five day-old male flies were starved for 6 h on agar only and then transferred to the vials containing agar, sucrose and 10 or 20 mM PQ for 12 and 24 h. The concentration of PQ was chosen according to a previous study in *Drosophila* by Chaudhuri et al. [6]. Control flies were transferred to vials having agar and sucrose, i.e., without the test chemical. All the chemicals of highest purity were obtained from Sigma Aldrich (St. Louis, MO, USA) unless otherwise stated.

### High-performance liquid chromatography (HPLC) analysis of PQ, dopamine (DA) and DOPAC

The HPLC analysis was performed using a Waters515series HPLC system (Water Milford, MA, USA). It consists of a binary pump, an on-line degasser, electro coupled and photo array detectors along with a reverse phased C-18 ODS analytical column (250 mm×4 mm; 5 µm particle size).

The level of PQ in the head of exposed *Drosophila* was estimated by following the method of Corasaniti and Nistico [32] with minor modifications. Tissue samples were homogenized in 0.1 M perchloric acid and centrifuged at 10000×g at 4°C. Supernatant from each sample was mixed with ammonium hydroxide and the content was passed through chromatographic column, prepared for ion-pair exchange and eluted with acidic methanol. The elute was evaporated to dryness and reconstituted in mobile phase consisting of 7.5 mM sodium heptanesulphonate and 0.1 M orthophosphoric acid (pH 3.0 with triethylamine) plus acetonitrile to yield a 10% (v/v) mixture. After injecting 50 µl sample in the HPLC injector, the level of PQ was estimated against a run time of 6 min (1.5 ml min^−1^ flow rate). Analysis was carried out using a Waters 2487 variable wavelength UV detector at 258 nm.

The levels of DA and DOPAC were measured by following the method of Yang and Beal [33] with minor modifications. Each sample was prepared from the dissected heads of 50–100 flies in a phosphate-buffered saline (1X PBS).The tissues were extracted with 0.1 M perchloric acid and then passed through 0.22 µm filter. Fifty microlitre extract was injected in the HPLC loop and separated on a mobile phase (0.1 M potassium phosphate, 10% methanol and 1 mM heptanesulphonic acid) with a flow rate of 1.0 ml min^−1^ against a run time of 10 min. Elutes from control and treated samples were analyzed using Waters 464 pulsed electrochemical detector against an appropriate standard.

### Assay of oxidative stress (OS) parameters

To estimate PQ-induced OS, level of superoxide radical (O_2_^−^), SOD activity, peroxynitrite anion (ONOO^−^) generation, and malondialdehyde (MDA) content were measured in the brain samples of control and PQ-exposed Drosophila.

Generation of superoxide (O_2_^−^) in the brain tissue of control and PQ-exposed groups was measured by flowcytometry using dihydroethidium dye (DHE; Invitrogen, USA) as described previously [34]. In brief, single-cell suspension was prepared from the dissected brain using collagenase (0.5 mg/ml) [30]. The cell suspension was incubated with DHE (10 µM) for 1 h in the dark at 24±1°C. After incubation, the cell suspension was washed with 1X PBS and finally, re-suspended in 1X PBS for analysis. The quantity of DHE oxidized to its fluorescent form, 2-hydroxyethidium (HE), was measured. HE fluorescence was quantified at an excitation/emission wavelength of 535/617 nm, using a Becton Dickinson flowcytometer (BD Biosciences, New Jersey, USA). O_2_^−^ level was measured using the mean fluorescence intensity of HE in each sample.

Superoxide dismutase (SOD) (superoxide: superoxide oxidoreductase EC 1.15.1.1) activity in the brain tissue of control and exposed organism was determined biochemically following the method described earlier [35] with minor modifications [15]. The reaction mixture was prepared as given in table 1.The reaction was started and stopped by using 780 µM reduced nicotinamide adenine dinucleotide (NADH) and glacial acetic acid (GAA) respectively. Finally, the coloured end product was extracted using n-butanol. Enzyme concentration required for 50% inhibition of chromogen production (optical density at 560 nm) was considered as one unit of enzyme activity. The results were expressed as specific activity in units min^−1^ mg^−1^ protein.

**Table 1:** Composition of reaction mixture for assaying SOD activity.

	Sodium pyrophosphate buffer (0.052M; pH 8.3)	Doubled distilled water	10% brain homogenate (w/v)	Nitroblue tetrazolium (300 µM)	Phenazine methosulphate (186 µM)	n-butanol
Blank	-	-	-	-	-	4.0 ml
Standard	1.2 ml	1.2 ml	-	300 µl	100 µl	4.0 ml
Sample	1.2 ml	1.175 ml	25 µl	300 µl	100 µl	4.0 ml

Peroxynitrite (ONOO^−^) production in Drosophila brain was monitored using dihydrorhodamine 123 (DHR 123; Cayman Chemical, MI, USA) as described previously [36] using a Becton Dickinson flowcytometer. The conversion of non-fluorescent DHR 123 to rhodamine, a fluorescent product after its oxidation with ONOO^−^, was measured at an excitation/emission wavelength of 500/536 nm. Briefly, single cell suspension from control and exposed groups was incubated with 20 µM DHR 123 for 10 min at 24±1°C. Thereafter, the cells were washed with 1X PBS and suspended in the same buffer before analysis. The mean fluorescence intensity was used as a measure of ONOO^−^ generation.

Generation of malondialdehyde (MDA), an intermediate during lipid peroxidation (LPO), was measured using tetraethoxypropane as an external standard [37]. The assay mixture was prepared using 25 µl of brain homogenate, 10% SDS, 20% acetic acid (pH 3.5) and 0.8% thiobarbituric acid (TBA). The mixture was kept in boiling water (100°C) for 1 h. Finally, the coloured end product was extracted using n-butanol. MDA content was expressed in terms of n moles MDA formed mg^−1^ protein after measuring the absorbance at 532 nm.

Protein content in brain sample of control and PQ-exposed groups were estimated by the method of Lowry et al [38], using bovine serum albumin (BSA) as the standard in range of 50–1000 µg/ml .

### Western blot analysis

Brain tissues from control and PQ-exposed flies were homogenized in a buffer (50 mM Tris-HCl; pH 7.5, 1 mM EGTA, 0.5 M NaCl, 1% Triton X-100, 1 mM DTT with protease inhibitors) [39]. Each tissue homogenate was centrifuged at 10000×g for 5 min at 4°C. The supernatant was used for protein estimation and western blotting. Quantification of protein in each sample was performed using Bradford's reagent in order to ensure equal loading. Protein samples (40 µg/lane) from control and exposed groups were separated by a 12.5% linear sodium dodecyl sulfate polyacrylamide gel electrophoresis (SDS-PAGE) at 15 mA. After electrophoresis, polypeptides were transferred to a polyvinylidene difluoride (PVDF) membrane (Amersham Pharmacia Biotech Limited, Buckinghamshire, England) using a semi-dry transfer method (TE70 PWR, Amersham Biosciences, NJ, USA). The membrane was incubated with TBST buffer (10 mM Tris-HCl, 150 mM NaCl, 0.1% Tween 20, pH 7.4) containing 5% non-fat milk for blocking non-specific binding of antibodies. After blocking, the membrane was probed with primary antibodies. Anti-Phospho-JNK, anti-cleaved caspase-3 (1∶1000; Cell Signaling Technology, MA, USA), anti-JNK (1∶500; Santa Cruz, TX, USA), Drosophila Hsp70 monoclonal antibody (7Fb; 1∶250) and anti-β tubulin (1∶500; Developmental Studies Hybridoma Bank, IA, USA) were used as the primary antibodies. For immuno-detection, Horse Radish Peroxidase conjugated secondary antibodies (1∶2000) were used. Finally, proteins were visualized using Femato reagent (Thermo Fisher Scientific, Rockford, USA) on a GelDoc (Bio-Rad, CA, USA).

### Assay of DEVDase (caspase 3-like) activity

The assay was performed according to the manufacturer's protocol (Bio Vision Inc. CA, USA). Briefly, the supernatant from 10% brain homogenate of control and PQ-exposed groups was mixed with chilled cell lysis buffer, 2X reaction buffer and 200 µM substrate. The reaction mixture was incubated at 37°C for 1.5 h and absorbance of colorimetric reaction was read at 405 nm on a Cintra 20 ultraviolet spectrophotometer (GBC Scientific Equipment, Melbourne, Australia). The DEVDase activity was calculated in terms of µmol pNA released min^−1^ mg^−1^ protein.

### Immunofluorescent analysis of dopaminergic neurons

Adult fly brains from control and PQ-exposed groups were fixed with cold 4% paraformaldehyde and dissected for immunostaining [40]. Brain tissues were washed thrice in PBST (1X PBS + 0.1% Triton X 100) and incubated overnight at 4°C in blocking solution [1X PBST + 5% heat inactivated fetal bovine serum (FBS)]. The tissues were then incubated over-night with primary antibody at 4°C. Rabbit anti-*Drosophila* Tyrosine Hydroxylase (1∶200; DTH) and Cy-3 conjugated goat anti-rabbit (1∶400) were used as the primary and secondary antibody, respectively. After staining, brain tissues were mounted in Vectashield mounting media (Vector Laboratories, CA, USA). Tissues were visualized under a Leica TCS-SPE confocal microscope (Nussloch, Germany) and images were captured at 200X. The numbers of DTH-positive neurons within each of the major dopaminergic neuron clusters were determined by visual examination of individual confocal Z-series images. Finally, the images were processed using Adobe Photoshop 7.0 software.

### Climbing assay

The locomotor performance of control and PQ-exposed flies was performed based on the negative geotaxis behavior, as described previously [41], [42] with minor modifications. In brief, 20 flies in a vertical plastic tube (18 cm×2 cm) were gently tapped. After 30 sec, flies those climbed over the 15 cm distance (n_top_) and those remaining below (n_bot_) that mark were scored separately. Ten trials were performed each at one min interval and three independent experiments were carried out for each genotype. For each experiment, using the formulae [1/2[(n_tot_+n_top_-n_bot_)/n_tot_], a performance index (PI) was calculated from the values obtained. Results were expressed as mean ± SD of the scores obtained from three independent experiments.

### Paraquat resistance test

Male *Drosophila* were separated at the pupal stage and after emergence, the flies were fed normal food for five days. Four hundred flies were starved for 6 h on 1.3% agar to ensure non-existence of food particles in their digestive tract and also to save them from dehydration. After fasting, 20 flies were kept in each vial containing filter papers soaked with 200 µl solution consisting of 5% sucrose and 20 mM PQ, while control flies received 5% sucrose only. Solutions were renewed every alternate day till the last fly died. Scoring of dead flies was performed after every 12 h.

### Statistical analysis

Analysis of all data was performed in a Prism software (GraphPad version 5.0, San Diego, CA, USA) using two-way analysis of variance (ANOVA) followed by Bonferani's test for multiple comparisons. After ascertaining the homogeneity of variance and normality of data, p<0.05 was considered as statistically significant. Pearson's correlation was calculated and then linear regression analysis was carried out. Using Kaplan–Meier's analysis with stratified log rank test by SPSS software version 13.0 (SPSS Inc., Chicago, USA), survival was assessed.

## Results

### Recovery of PQ from the exposed Drosophila

We detected ∼2.68–2.98 µM PQ in the head of flies exposed to 20 mM PQ for 24 h. However, in the head of control flies, PQ level was beyond the limit of detection (Table 2). An insignificant difference was observed in the amount of PQ recovered from each genotype at both the exposure concentrations (10 and 20 mM) (Table 2).

**Table 2:** PQ estimation in the head of different fly strains after 24

	*w^1118^*	*Df(hsp70)*	*TH-Gal4>w^1118^*	*TH-Gal4>UAS-hsp70*	*TH-Gal4>HSP70K71E*	*TH-Gal4>HSPA1L*
0 mM	ND	ND	ND	ND	ND	ND
10 mM	1.76±0.23	1.85±0.21	1.69±0.25	1.72±0.18 2.68±	1.75±0.22	1.67±0.15
20 mM	2.64±0.25	2.77±0.18	2.98±0.14	0.26	2.86±0.24	2.74± 0.21

Data represent mean ± SD of three identical experiments made in triplicates; values were represented in µM. ND: not detected.

### *hsp70* over-expression diminished PQ-induced OS along with JNK and caspase-3 activation

**Figure 1:**
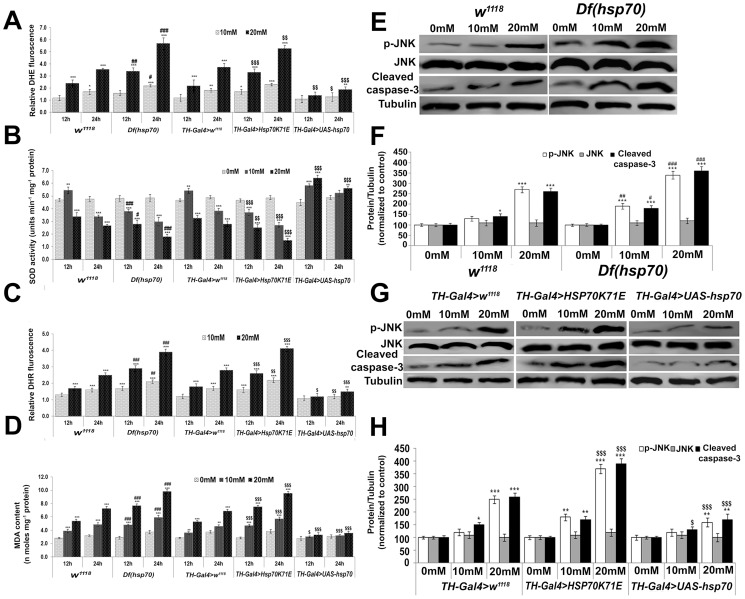
*hsp70* over-expression in the dopaminergic neurons of *Drosophila* protect them from PQ-induced OS. (A) Time and dose dependent increase in superoxide (O_2_^−^) in the brain of PQ-exposed *w^1118^*, *Df(hsp70)*, *TH-Gal4>w^1118^* and *TH-Gal4>Hsp70K71E* flies and less generation of O_2_^−^ in exposed *TH-Gal4>UAS-hsp70* flies as compared to *TH-Gal4>w^1118^.* (B) Decreased SOD activity in the brain of PQ-exposed *w^1118^*, *Df(hsp70), TH-Gal4>w^1118^* and *TH-Gal4>Hsp70K71E* flies and an increased enzyme activity in exposed *TH-Gal4>UAS-hsp70* flies. (C) Graphical representation of peroxynitrite (ONOO^−^) generation using DHR 123 staining. ONOO^−^ generation was also found to be less like O_2_^−^ generation in PQ-exposed *TH-Gal4>UAS-hsp70* flies. (D) Histogram depicting the extent of lipid peroxidation as determined by MDA content in *w^1118^*, *Df(hsp70)*, *TH-Gal4>Hsp70K71E* and *TH-Gal4>UAS-hsp70* flies. (E) Representative immuno-blot images of p-JNK, JNK and cleaved caspase-3 levels in the protein samples from brain tissues of control and PQ-exposed *w^1118^* and *Df(hsp70)* flies. (F) Densitometry analysis of data normalized against loading control tubulin. (G) Representative immuno-blotting images of *TH-Gal4>w^1118^*, *TH-Gal4>HSP70K71E* and *TH-Gal4>UAS-hsp70* flies. (H) Densitometry analysis of data normalized against loading control tubulin. Values are mean ± SD (n = 3). Significance were ascribed as *p<0.05, **p<0.01 and ***p<0.001 vs. control; ^$^ *p*<0.05, **^$$^** *p*<0.01 and **^$$$^** *p*<0.001 vs. *TH-Gal4>w^1118^*; **^#^** *p*<0.05, **^##^** *p*<0.01 and **^###^** *p*<0.001 vs. *w^1118^*.

Prior to experiments, stocks were validated by RT-PCR using gene specific primers (Figure S1). We observed significantly less (∼1.8 fold) generation of O_2_^−^ in the brain of PQ-exposed flies (20 mM for 24 h) that over-expressed hsp70 in their dopaminergic neurons (TH-Gal4>UAS-hsp70) as compared to similarly exposed TH-Gal4>w^1118^ flies (Figure 1A). Conversely, similar exposure to Df(Hsp70) and TH-Gal4>HSP70K71E flies resulted in ∼5.7 and ∼5.3 fold increase in O_2_^−^ generation respectively as compared to respective controls (Figure 1A). Further, significantly decreased SOD activity was observed in the brain of 20 mM PQ-exposed w^1118^, Df(Hsp70), TH-Gal4>HSP70K71E and TH-Gal4>w^1118^ flies for 24 h, while similarly exposed TH-Gal4>UAS-hsp70 flies showed significantly increased SOD activity (Figure 1B). Concomitant with increased SOD activity and less generation of O_2_^−^ in 20 mM PQ-exposed TH-Gal4>UAS-hsp70 flies, significantly lower ONOO^−^ generation (∼1.3 fold) was observed in their brain as compared to respective TH-Gal4>w^1118^ (Figure 1C). However, a time- and concentration-dependent increase in ONOO^−^ generation was observed in the brain of PQ-exposed w^1118^, Df(Hsp70), TH-Gal4>HSP70K71E and TH-Gal4>w^1118^ flies in comparison to respective controls (Figure 1C). We also observed significantly lower (∼0.7 fold) MDA content in exposed TH-Gal4>UAS-hsp70 flies as compared to TH-Gal4>w^1118^ flies (Figure 1D). Conversely, significantly higher MDA content was observed in the brain of PQ-exposed w^1118^, Df(Hsp70) TH-Gal4>HSP70K71E and TH-Gal4>w^1118^ flies in comparison to their respective controls (Figure 1D).

**Figure 2:**
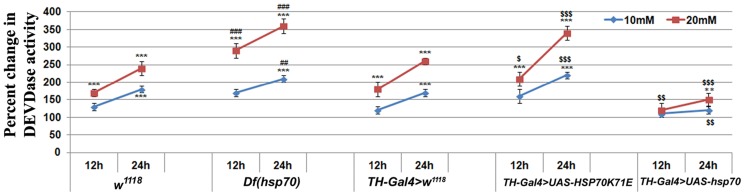
DEVDase (caspase 3-like) activity in the brain of PQ-exposed *Drosophila*. Graphical representation of DEVDase activity in the brain homogenate of *Drosophila* after their exposure to PQ for 12 and 24 h; A significant decrease in enzyme activity was observed in the brain of PQ-exposed *hsp70* over-expressing strain. Data are mean ± SD (n = 3). Significance ascribed as **p<0.01 and ***p<0.001 vs. control; **^$^** *p*<0.05; **^$$^** *p*<0.01 and **^$$$^** *p*<0.001 vs. *TH-Gal4>w^1118^*; **^##^** *p*<0.01 and **^###^** *p*<0.001 vs. *w^1118^*.

Along with reduced OS in exposed TH-Gal4>UAS-hsp70 flies, the levels of p-JNK and cleaved caspase-3 were found to be significantly lower in the brain of these organisms as compared to respective TH-Gal4>w^1118^ (Figure 1G–H). A similar trend was also observed for DEVDase activity (Figure 2). Unlike the above, we observed significantly higher levels of p-JNK and cleaved caspase-3 along with increased DEVDase activity in the brain of PQ-exposed w^1118^, Df(Hsp70) and TH-Gal4>HSP70K71E flies (Figure 1E–H; Figure 2) in comparison to respective controls.

### *hsp70* over-expression protects dopaminergic neuronal degeneration

**Figure 3:**
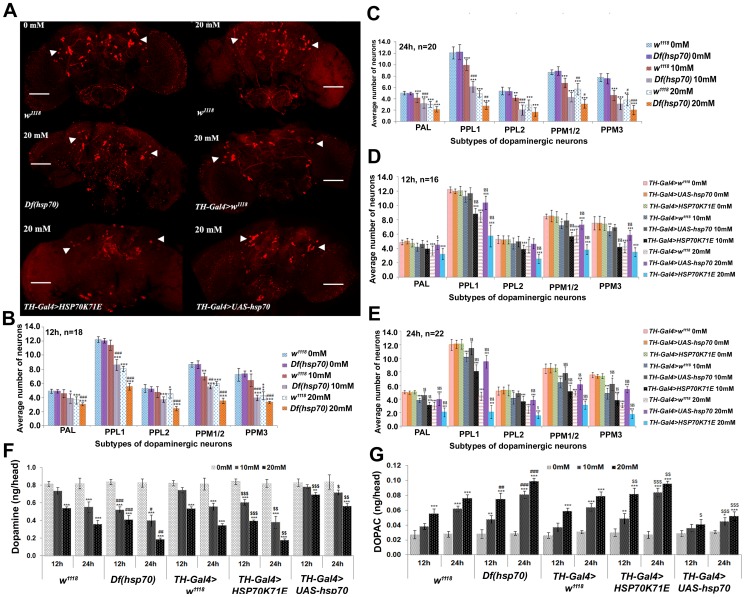
*hsp70* over-expression in the dopaminergic neurons of *Drosophila* protects them from PQ-induced neuronal degeneration. (A) Representative confocal images of brains of control *w^1118^* and 20 mM PQ-exposed *w^1118^*, *Df(hsp70)*, *TH-Gal4>w^1118^*, *TH-Gal4>HSP70K71E* and *TH-Gal4>UAS-hsp70* fly for 24 h after anti-DTH staining. Quantitative representation of the average number of neurons in the dopaminergic clusters of adult flies that were exposed to PQ (B–D) for 12 and (C–E) 24 h. Graphical representation of (F) DA and (G) DOPAC levels in PQ-exposed *w^1118^*, *Df(hsp70)*, *TH-Gal4>w^1118^*, *TH-Gal4>HSP70K71E* and *TH-Gal4>UAS-hsp70* flies. Arrow head indicates PPL1 cluster. Significance ascribed as *p<0.05, **p<0.01 and ***p<0.001 vs. control; **^$^** *p*<0.05, **^$$^** *p*<0.01 and **^$$$^** *p*<0.001 vs. *TH-Gal4>w^1118^*; **^#^** *p*<0.05, **^##^** *p*<0.01 and **^###^** *p*<0.001 vs. *w^1118^*. Bar = 20 µm

Concurrent with lower OS and less JNK activation observed in the brain of PQ-exposed *hsp70* over-expressing flies, we also observed significantly less neurodegeneration in these organisms (Figure 3A; 3D–E). However, an extensive loss of dopaminergic neurons occurred in PQ-exposed *Df*(*Hsp70*) and *TH-Gal4>HSP70K71E* flies (Figure 3A–E) as compared to respective *w^1118^* and *TH-Gal4>w^1118^* flies. In addition, we observed a non-significant difference in the dopaminergic neuronal degeneration among PQ-exposed Gal4- or UAS- alone flies (Figure S2). After analysis, PPL1 cluster was found to be the most sensitive cluster as compared to others, which is parallel to a previous report [6]. In this context, *w^1118^* flies that were exposed to 20 mM PQ for 24 h, exhibited ∼61% neuronal degeneration in the PPL1 cluster while in similarly exposed *Df(hsp70)* and *TH-Gal4>HSP70K71E* flies, neurodegeneration was ∼77 and ∼83% respectively (Figure 3C; 3E). In PQ-exposed *TH-Gal4>UAS-hsp70* flies, only ∼22% neurodegeneration was observed in their PPL1 cluster (Figure 3E). The observed protection of dopaminergic neurons in PQ-exposed *hsp70* over-expressing flies was further supported by significantly less decline in DA and lowered DOPAC levels as against respective *TH-Gal4>w^1118^* flies (Figure 3F–G). However, flies with a *hsp70* deficiency [*Df(hsp70)*] or diminished Hsp70 level [*TH-Gal4>HSP70K71E*] showed further loss of DA and higher DOPAC level as compared to similarly exposed *w^1118^* and *TH-Gal4>w^1118^* flies (Figure 3F–G).

### Improved locomotor performance and survival benefit in PQ-exposed *hsp70* over-expressing *Drosophila*

**Figure 4:**
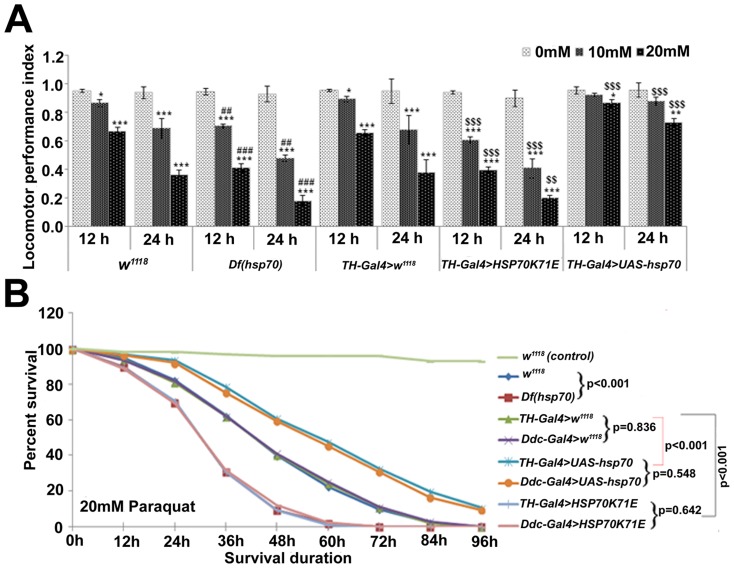
*hsp70* over-expression protects PQ-exposed flies from impaired locomotor performance and poor survival. (A) Graphical representation of the negative geotaxis assay performed in PQ-exposed *w^1118^*, *Df(hsp70)*, *TH-Gal4>w^1118^*, *TH-Gal4>HSP70K71E* and *TH-Gal4>UAS-hsp70* flies after their exposure to PQ. (B) Survival curve representing pooled data of five independent replicate experiments. Values are mean ± SD (n = 5). Significance ascribed as *p<0.05, **p<0.01 and ***p<0.001 vs. control; **^$$^** *p*<0.01 and **^$$$^** *p*<0.001 vs. *TH-Gal4>w^1118^*; **^##^** *p*<0.01 and **^###^** *p*<0.001 vs. *w^1118^*.

To examine the behavioral response as a measure of PQ-induced PD like symptoms in exposed Drosophila, we monitored locomotor performance of flies that were exposed to PQ. Nearly 64% of PQ-exposed w^1118^ flies (20 mM PQ for 24 h) failed to cross the 15 cm mark within 30 sec while ∼85% of exposed Df(hsp70) flies met the same fate (Figure 4A). The latter group of flies even failed to cross the stipulated height after 90 sec. Conversely, improved locomotor behavior was observed in exposed hsp70 over-expressing flies, where only ∼31% of them failed to cross the above mark in stipulated time.

The benefit of over-expressing hsp70 in the dopaminergic neurons in exposed organism could be weighed by the survival advantage gained by these flies (∼33 and ∼11% fly survived after 72 and 96 h of 20 mM PQ exposure respectively) as against respective TH-Gal4>w^1118^ flies (only ∼11% survived after 72 h exposure period while none after 96 h) (Figure 4B).

### Targeted expression of *HSPA1L* in dopaminergic neurons protects the organism from PQ-induced PD like symptoms

**Figure 5:**
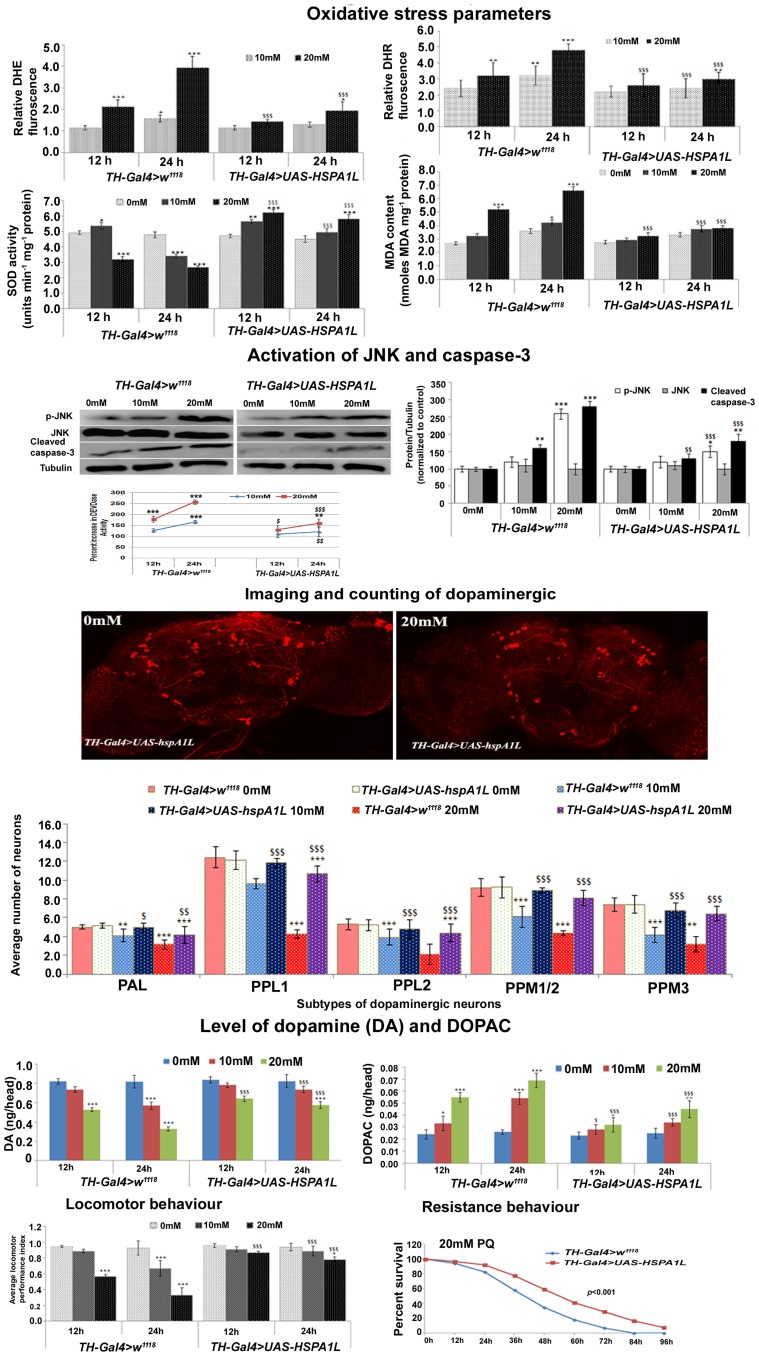
Over-expression of a human homologue of *hsp70* in the dopaminergic neurons of *Drosophila* provided them resistance against PQ-induced PD like symptoms. Like PQ-exposed *TH-Gal4>UAS-hsp70* flies, exposed *TH-Gal4>UAS-HSPA1L* flies also exhibited reduced levels of OS as evidenced by less O_2_^−^ generation, improved SOD activity, less formation of ONOO^−^ and non-significant increase in MDA content. JNK phosphorylation and cleaved caspase-3 level was significantly less in the brain of PQ-exposed *TH-Gal4>UAS-HSPA1L* flies in comparison to Gal4 control, as represented by immune-blotting images and densitometry analysis. Dopaminergic neuronal health depicted by representative confocal image of anti-DTH stained brain of control and PQ-exposed *TH-Gal4>UAS-HSPA1L* flies and graphical representation of dopaminergic neurons in different clusters (n = 22). In the brain of PQ-exposed *TH-Gal4>UAS-HSPA1L* flies, less decline in DA and lower level of DOPAC was observed. Improved locomotor performance and survival advantage were also observed in these flies. Values are mean ± SD (n = 3). Significance ascribed as *p<0.05, **p<0.01 and ***p<0.001 vs. control; ^$^ *p*<0.05, ^$$^ *p*<0.01 and ^$$$^ *p*<0.001 vs. *TH-Gal4>w^1118^*.

To examine whether over-expression of a human homologue of hsp70 (HSPA1L) in the dopaminergic neurons of PQ-exposed flies can resist PD like symptoms in a way similar to that observed in Drosophila hsp70 over-expressing strain, we investigated the above endpoints. PQ-exposed TH-Gal4>UAS-HSPA1L flies exhibited significantly less generation of reactive species (O_2_^−^, ONOO^−^), increased SOD activity, improved dopaminergic neuronal health and locomotor performance, less activation of apoptotic signalling along with their improved survival in comparison to similarly exposed TH-Gal4>w^1118^ (Figure 5).

## Discussion

Considering the anti-apoptotic function of Hsp70 and association of PQ exposure with PD, the results of the present study provide evidences for the first time on the protective role of Hsp70 against PQ-induced PD like symptoms in exposed Drosophila. The protective mechanism of Hsp70 against PQ-induced neurodegeneration was observed in terms of reduced OS, stalled activation of JNK and caspase-3 mediated dopaminergic neuronal apoptosis in exposed organism.

A non-significant difference in the amount of PQ recovered from different genotypes of exposed Drosophila suggests a nearly equivalent feeding pattern of all tested strains. The amount of PQ recovered supports the underlying mechanism of poor absorption and localization of the PQ in Drosophila brain similar to mammalian models [43], [44].

The adverse effect of PQ is reported to be associated with free radical generation [11] especially superoxide radical (O_2_^−^), which is dismutated to hydrogen peroxide (H_2_O_2_) by an anti-oxidant defence enzyme, SOD, in the cellular system [45]. Since an imbalance between reactive oxygen species (ROS) generation and anti-oxidant defence leads to OS, decreased SOD activity and increased O_2_^−^ generation can enhance OS in PQ-exposed organism. A negative co-relation drawn between hsp70 expression and O_2_^−^ generation while a positive co-relation drawn between SOD activity and hsp70 expression indicates the possible protective role of Hsp70 against PQ-induced OS. Interestingly, we observed decreased SOD activity in the brain of PQ-exposed w^1118^, Df(hsp70) and TH-Gal4>HSP70K71E flies along with generation of higher level of O_2_^−^. Since O_2_^−^ is a substrate of SOD, higher level of O_2_^−^ may lead to increased SOD activity in w^1118^, Df(hsp70) and TH-Gal4>HSP70K71E flies. Instead, decreased SOD activity was observed in these organisms which is explained in the following: The reaction between O_2_^−^ and nitric oxide (NO) generates peroxynitrite (ONOO^−^) anion in a cell causing nitration of SOD which can diminish its activity [36], [46]. Further, reciprocal relation observed between ONOO^−^ generation and SOD activity in the brain of PQ-exposed organism provides a plausible explanation for the decreased SOD activity in these organisms. Since LPO is considered as one of the mechanisms of PQ toxicity [47], decreased LPO in the brain of PQ-exposed hsp70 over-expressing flies suggests the protective role of Hsp70 against PQ-induced OS. Our observation finds support from an earlier study wherein lifelong over-expression of hsp70 in the skeletal muscle of mice provided them protection against age-related OS through the proper functioning of anti-oxidant system [48].

PQ-induced OS has been shown to be linked with neuronal cell death by JNK phosphorylation and caspase-3 activation [7] which was supported by our observations in PQ-exposed w^1118^ and Df(hsp70) flies. However, diminished JNK phosphorylation and caspase-3 activation observed in PQ-exposed hsp70 over-expressing flies is in agreement with the anti-apoptotic role of Hsp70 [49], [50]. Since JNK activation and apoptosis are considered as one of the principal mechanisms of PQ-induced neurodegeneration [7], blockage of these events by hsp70 over-expression suggests better survival of neuronal cells against PQ exposure. Given that hsp70 was found to be non-significantly induced in the brain of PQ-exposed w^1118^ flies (Figure S3), over-expression of hsp70 in the brain of exposed organism is likely to counteract PQ-induced cell death by virtue of its anti-apoptotic and anti-oxidative properties.

The observed protection achieved in hsp70 over-expressing flies against OS and JNK activation was confirmed by improved dopaminergic neuronal health in these organisms. Dopaminergic neuronal cell death has been considered as the pathological hallmark of PD [51] which is manifested by a decline in DA and an elevation of DOPAC level. In this context, increased ONOO^−^ generation in exposed organism may cause nitration of TH, a key enzyme in DA synthesis [52] which may inactivate it suggesting a negative role of the anion on enzymatic activity of TH. In addition, increased ONOO^−^ generation was in parallel to depleted DA content along with degeneration of dopaminergic neurons in PQ-exposed w^1118^, Df(hsp70) and TH-Gal4>HSP70K71E flies. Reversal of the above findings in hsp70 over-expressing flies established a neuro-protective role of Hsp70 against PQ insult in exposed organism.

Less degeneration of dopaminergic neurons and better locomotor performance in PQ-exposed hsp70 over-expressing flies observed in this study substantiate the earlier observed co-relation between dopaminergic neuronal degeneration and locomotor performance in different models [6], [53]. Thus, from both gain- as well as loss-of-function data, it is apparent that Hsp70 can rescue PQ-induced neurodegeneration by modulating OS induced activation of JNK and caspase-3 mediated cell death. The observed protection was further substantiated by improved survival of exposed hsp70 over-expressing organism. Two Gal4 lines (Ddc and TH) were used for manipulation of gene expression in dopaminergic neurons of Drosophila [54], [55]. We observed almost similar trend in the survival of PQ-exposed hsp70 over-expressing flies (TH-Gal4>UAS-hsp70 and Ddc-Gal4>UAS-hsp70) as compared to respective Gal4 control and w^1118^ flies suggesting Gal4 independent effect of Hsp70 (Figure 4B). Our observation finds support from a previous study by Azad et al. [56], wherein survival advantage against hypoxia was demonstrated by over-expressing hsp70 in the brain (c739-Gal4) and lymph gland (hand-Gal4) of flies. In addition, less generation of free radicals in the brain of PQ-exposed hsp70 over-expressing flies and a negative co-relation drawn between free radical generation and survival (Table 3) suggest that improved survival of these organisms was due to lower generation of reactive species. This is a consistent observation which is supported by an earlier report, wherein survival of the organism was shown to be affected by free radicals [57].

**Table 3:** Correlation among ROS generation and % survival in PQ-exposed *Drosophila.*

	% survival	O_2_^−^	ONOO^−^
**% survival**	1		
**O_2_^−^**	−0.99681**	1	
**ONOO^−^**	−0.99378**	0.985479**	1

Negative correlation was observed between generation of ROS (O_2_^−^/ONOO^−^) and percent survival in different strains of *Drosophila* exposed to 20 mM PQ for 24 h. n = 4 degree of freedom; ** *p*<0.01.

The study assumes significance in context of human relevance since a comparable protection against PD like symptoms in PQ-exposed organism was observed using both Drosophila hsp70 and its human homologue (HSPA1L) suggesting the therapeutic potential of Hsp70 in neurodegenerative disorders caused by environmental chemicals.

## Conclusions

**Figure 6:**
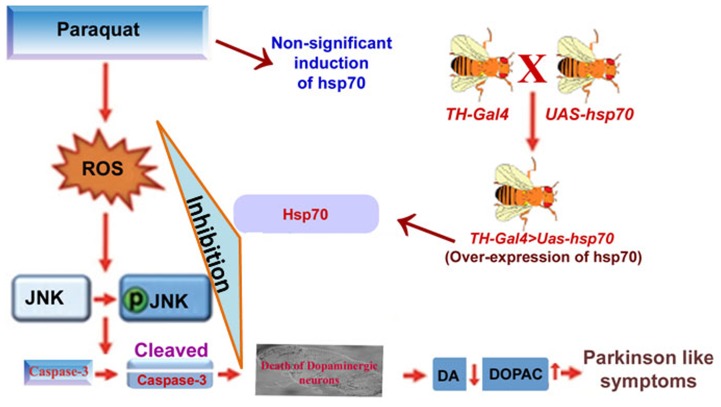
Schematic representation of the neuro-protective role of Hsp70 against PQ-induced PD like symptoms. PQ-induced ROS generation led to the activation of JNK and caspase-3, which eventually caused death of the dopaminergic neurons and induces PD like symptoms in the exposed organism. The observed PD like symptoms was resisted by over-expression of one of the key chaperons, Hsp70, in the dopaminergic neurons of *Drosophila*. Hsp70 inhibits PQ-induced activation of JNK and caspase-3 mediated neuronal cell death, thereby providing protection to the organism.

The study demonstrated that hsp70 over-expression (both Drosophila and its human homologue) in the dopaminergic neurons of Drosophila protects the organisms from PQ-induced PD like symptoms as evidenced by improved dopaminergic neuronal health, better locomotor performance and improved survival. PQ-induced adversities, mainly governed by the generation of reactive species (O_2_^−^ and ONOO^−^), was found to be responsible for the phosphorylation of JNK and activation of caspase-3 mediated dopaminergic neuronal cell death in exposed organism (Figure 6). In conclusion, we have presented the possible mechanism of protection against PQ-induced PD like symptoms in exposed organism using a key molecular chaperone, Hsp70. Further, the study recommends the use of Drosophila as an in vivo model for the evaluation of potential therapeutic targets against environmental chemicals induced disease like condition with minimum ethical concern.

## Supporting Information

Figure S1 ***hsp70*** **expression in the brain of** ***Drosophila*** **.** For fly stock validation genotypically, total RNA was isolated from the brain of five-day-old flies using TRI reagent (Ambion, Austin, TX, USA), chloroform, isopropanol and ethanol following the manufacturer's instructions. The isolated RNA was used for cDNA synthesis using Revert Aid H Minus first strand cDNA synthesis kit (Fermentas MD, USA) according to the manufacturer's protocol. Each reaction mixture consisted of total RNA, 0.5 µg/µl oligo (dT)_18_ primer, 5x reaction buffer, 20 U Ribolock ribonuclease inhibitor, 10.0 mM dNTP mixture, 200 U Revert Aid H Moloney Murine Leukemia Virus reverse transcriptase (M-MuLV RT) and DEPC water to make a final volume of 20 µl. The cDNA was amplified by PCR on a thermocycler (Eppendorf, Hamburg, Germany) using gene specific primers (Table S1). PCR reaction mixture (total 25 µl) consisted of 1X Taq buffer, 1.5 mM MgCl_2_, 0.20 mM dNTPs mixture, 0.40 µM each of forward and reverse primer, 1U Taq DNA polymerase (Fermentas Life Sciences, MD, USA), 2 µl cDNA and milli-Q water. The amplicons were separated on an 1.5% agarose gel containing ethidium bromide at 5 V/cm and visualized with a VERSA DOC Imaging System Model 1000 (Bio-Rad, CA, USA). The intensity of the band was quantified by Quantity One software (Bio-Rad, CA, USA). Each experiment was carried out thrice with three independent biological replicates. Glyceraldehyde 3-phosphate dehydrogenase (*GAPDH*) was used as an endogenous control. Representative agarose gel picture showing the expression of *hsp70* (A). Lanes 1-7 (L-R): Marker; no template control (NTC); *w^1118^*; *Df(hsp70)*; *TH-Gal4>w^1118^*; *TH-Gal4>UAS-hsp70* and *TH-Gal4>HSP70K71E*. (B) Agarose gel picture showing expression of *HSPA1L*. Lanes 1-4 (L-R): Marker; NTC; *TH-Gal4>w^1118^* and *TH-Gal4>UAS-HSPA1L*.
(TIF)

Figure S2 A non-significant difference in the dopaminergic neuronal degeneration between Gal4 and UAS construct bearing flies that were exposed to PQ. Confocal images and neuronal cell counts in the PPL1 cluster of *Drosophila* exposed to 20 mM PQ for 24 h, as revealed by anti-DTH staining. Magnification: 400X.
(TIF)

Figure S3 **PQ exposure to** ***w^1118^*** **flies resulted in non-significant induction of** ***hsp70*** **in their brain.** Hsp70 level as revealed by immune-blotting of protein samples prepared from the brain tissues of PQ-exposed *Drosophila*. For positive control (PC), flies were given temperature shock (37°C) for 1 h in a moistened glass vial. Flies were then allowed to recover at 25°C for 30 min before the sample preparation. A non-significant difference (*p*>0.05) in the level of Hsp70 was observed in the flies that were exposed to 20 mM PQ for 24 h as compared to unexposed flies.
(TIF)

Table S1 **Genes and their primer sequences used for PCR amplification.**
(DOC)
