# Supplementary figures and images for: Heat Shock Protein-70 (Hsp-70) Suppresses Paraquat-Induced Neurodegeneration by Inhibiting JNK and Caspase-3 Activation in Drosophila Model of Parkinson's Disease
Source: PLoS One. 2014 Jun 2;9(6):e98886. doi: 10.1371/journal.pone.0098886 (PMC4041817; doi:10.1371/journal.pone.0098886)

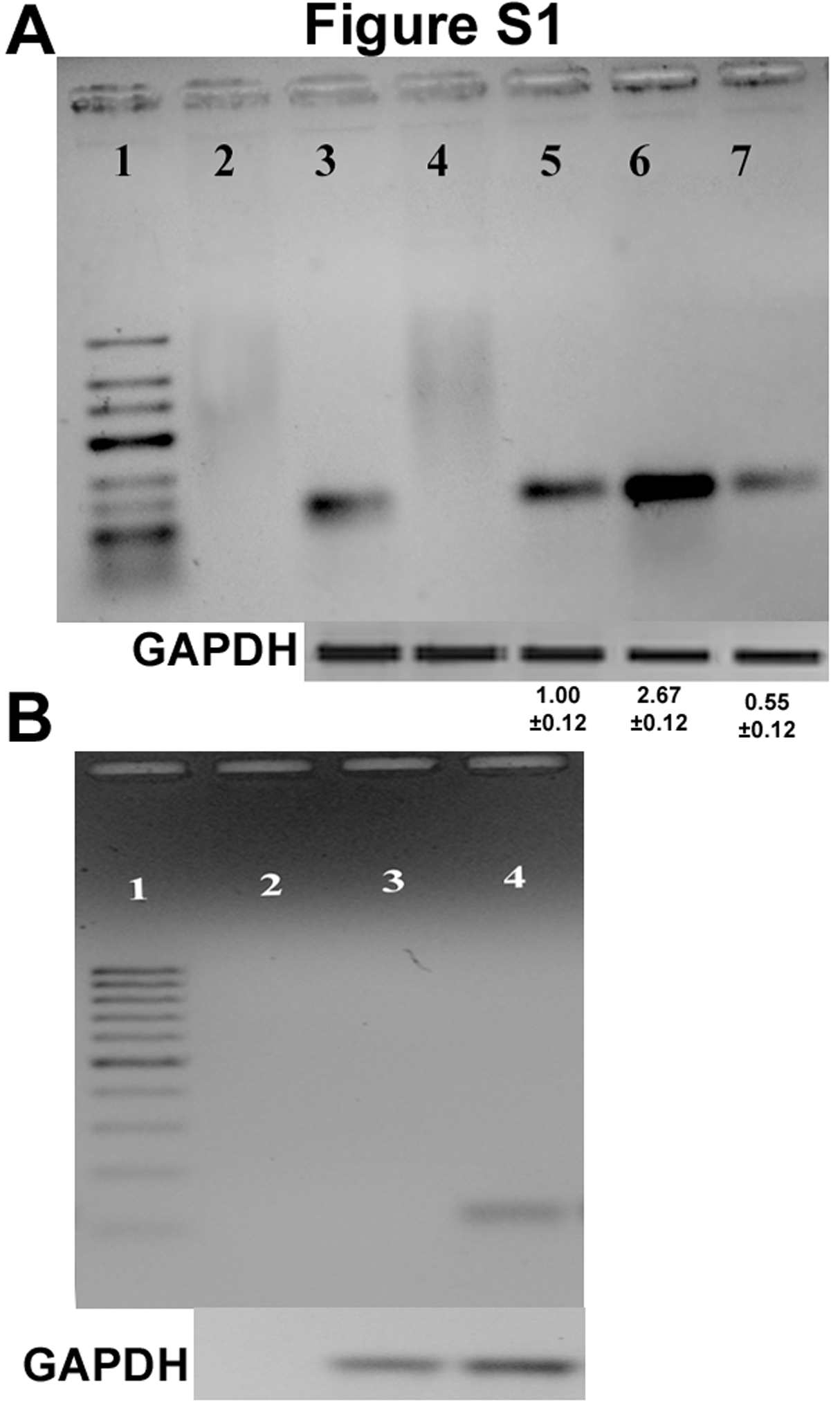

Supplement: Figure S1 — hsp70 expression in the brain of Drosophila . For fly stock validation genotypically, total RNA was isolated from the brain of five-day-old flies using TRI reagent (Ambion, Austin, TX, USA), chloroform, isopropanol and ethanol following the manufacturer's instructions. The isolated RNA was used for cDNA synthesis using Revert Aid H Minus first strand cDNA synthesis kit (Fermentas MD, USA) according to the manufacturer's protocol. Each reaction mixture consisted of total RNA, 0.5 µg/µl oligo (dT)18 primer, 5x reaction buffer, 20 U Ribolock ribonuclease inhibitor, 10.0 mM dNTP mixture, 200 U Revert Aid H Moloney Murine Leukemia Virus reverse transcriptase (M-MuLV RT) and DEPC water to make a final volume of 20 µl. The cDNA was amplified by PCR on a thermocycler (Eppendorf, Hamburg, Germany) using gene specific primers (Table S1). PCR reaction mixture (total 25 µl) consisted of 1X Taq buffer, 1.5 mM MgCl2, 0.20 mM dNTPs mixture, 0.40 µM each of forward and reverse primer, 1U Taq DNA polymerase (Fermentas Life Sciences, MD, USA), 2 µl cDNA and milli-Q water. The amplicons were separated on an 1.5% agarose gel containing ethidium bromide at 5 V/cm and visualized with a VERSA DOC Imaging System Model 1000 (Bio-Rad, CA, USA). The intensity of the band was quantified by Quantity One software (Bio-Rad, CA, USA). Each experiment was carried out thrice with three independent biological replicates. Glyceraldehyde 3-phosphate dehydrogenase (GAPDH) was used as an endogenous control. Representative agarose gel picture showing the expression of hsp70 (A). Lanes 1-7 (L-R): Marker; no template control (NTC); w1118; Df(hsp70); TH-Gal4>w1118; TH-Gal4>UAS-hsp70 and TH-Gal4>HSP70K71E. (B) Agarose gel picture showing expression of HSPA1L. Lanes 1-4 (L-R): Marker; NTC; TH-Gal4>w1118 and TH-Gal4>UAS-HSPA1L. (TIF) [file pone.0098886.s001.tif]

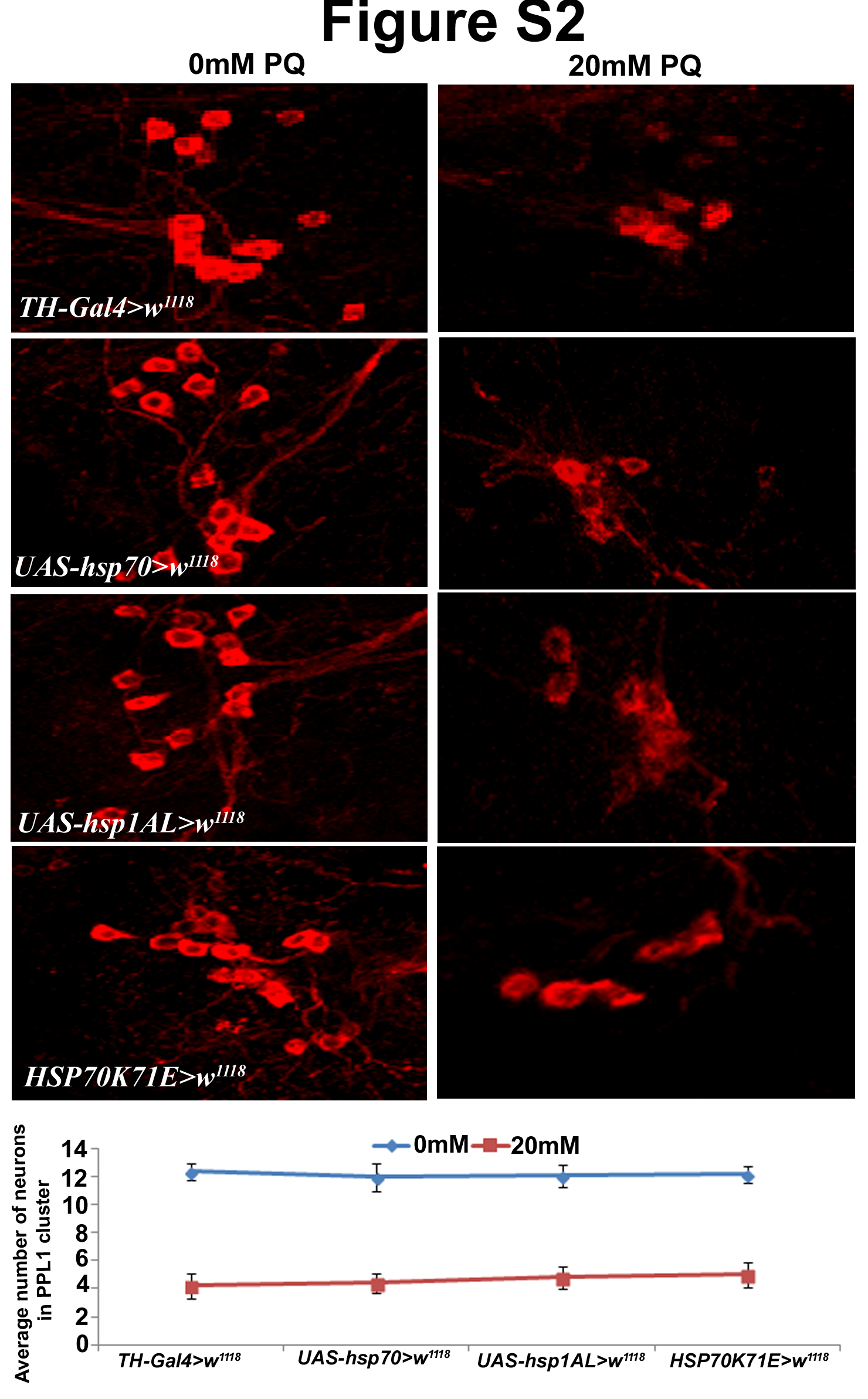

Supplement: Figure S2 — A non-significant difference in the dopaminergic neuronal degeneration between Gal4 and UAS construct bearing flies that were exposed to PQ. Confocal images and neuronal cell counts in the PPL1 cluster of Drosophila exposed to 20 mM PQ for 24 h, as revealed by anti-DTH staining. Magnification: 400X. (TIF) [file pone.0098886.s002.tif]

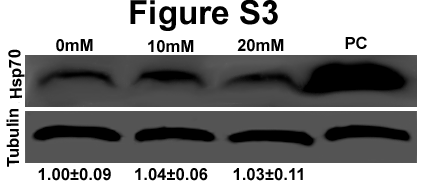

Supplement: Figure S3 — PQ exposure to w1118 flies resulted in non-significant induction of hsp70 in their brain. Hsp70 level as revealed by immune-blotting of protein samples prepared from the brain tissues of PQ-exposed Drosophila. For positive control (PC), flies were given temperature shock (37°C) for 1 h in a moistened glass vial. Flies were then allowed to recover at 25°C for 30 min before the sample preparation. A non-significant difference (p>0.05) in the level of Hsp70 was observed in the flies that were exposed to 20 mM PQ for 24 h as compared to unexposed flies. (TIF) [file pone.0098886.s003.tif]
