# Supplementary material for: Heat Shock Protein-70 (Hsp-70) Suppresses Paraquat-Induced Neurodegeneration by Inhibiting JNK and Caspase-3 Activation in Drosophila Model of Parkinson's Disease
Source: PLoS One. 2014 Jun 2;9(6):e98886. doi: 10.1371/journal.pone.0098886 (PMC4041817; doi:10.1371/journal.pone.0098886)
Supplement: Table S1 — Genes and their primer sequences used for PCR amplification. (DOC) [file pone.0098886.s004.doc]

**Table S1:** Genes and their primer sequences used for PCR amplification.

| **Genes** | **Primer sequences** |
| --- | --- |
| *hsp70* | F- 5ˈGAACGGGCCAAGCGCACACTCTC3ˈ  R- 5ˈTCCTGGATCTTGCCGCTCTGGTCTC3ˈ |
| *HspA1L* | F- 5ˈCAAACTGAAGCGAAGGCGTC3ˈ R- 5ˈATGATCTCCACCTTGCCGTG3ˈ |
| *GAPDH* | F- 5ˈAATTCCGATCTTCGACATGG 3ˈ  R- 5ˈGAAAAAGCGGCAGTCGTAAT3ˈ |
